# How I manage intracranial hypertension

**DOI:** 10.1186/s13054-019-2529-z

**Published:** 2019-07-04

**Authors:** Chiara Robba, Giuseppe Citerio

**Affiliations:** 1 Anaesthesia and Intensive Care, San Martino Policlinico Hospital, IRCCS for Oncology and Neuroscience, Genoa, Italy; 2 School of Medicine and Surgery, University of Milan-Bicocca, Milan, Italy; 3 Neurointensive Care Unit, San Gerardo Hospital, ASST-Monza, Monza, MB Italy

## Why and when to manage intracranial hypertension

The detrimental effects of intracranial hypertension (HICP, high intracranial pressure) are well documented [1, 2]. HICP can cause secondary brain injury and death, and therefore, intracranial pressure (ICP) elevations should be aggressively treated.

HICP has been classically defined as an ICP > 20 mmHg, and this threshold has been considered the trigger for treatment [3]. Recent BTF guidelines have moved this threshold to 22 mmHg [4], grounded on a single-centre, retrospective study. This modification is trivial [5]. As for many other treatment options in intensive care, a single threshold is debatable. In fact, recent evidence suggests that not a single value but the time spent over the threshold and its intensity, the so-called ICP dose, is more important [6]. Moreover, Guiza demonstrated that not only higher values but also prolonged exposure to values below the classical threshold are associated with negative outcomes [7]. In addition, if cerebral perfusion pressure (CPP, i.e. MAP-ICP) is critically low (< 50 mmHg), ICP is no longer a predictor for poor outcome and lower ICP values might be barely tolerated. On the contrary, ICP insults in the range 18–23 mmHg can be tolerated for a longer duration at higher CPPs. In my practice, the ICP alarm is set at 20 mmHg and low CPP alarm at 55 mmHg. This is a warning signal for nurses at the bedside. Before starting any treatments for high ICP, I consider both the intensity and duration of HICP. I am flexible with thresholds putting them in the clinical contest, considering also CPP. Short-lasting, low-intensity episodes (low ICP dose with normal CPP) are observed and not treated. On the contrary, higher ICP doses, progressively rising trends, or/and HICP impacting CPP require prompt treatment.

## How I manage intracranial hypertension

**Fig. 1:**
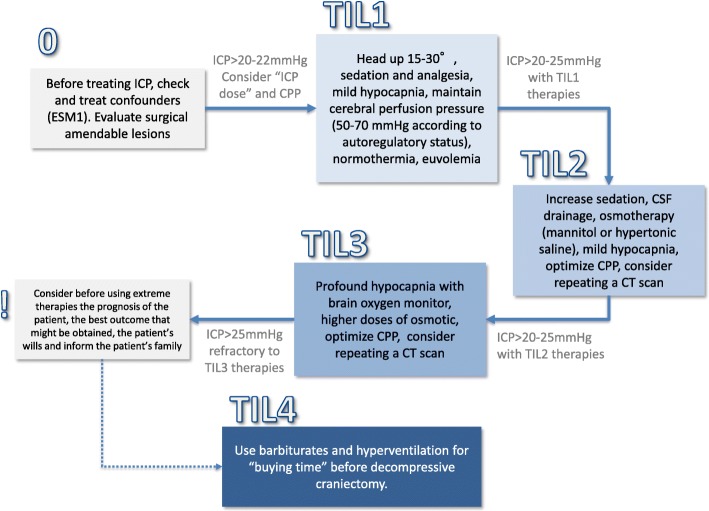
Summary of the available ICP-directed therapies. Before starting an HICP-directed therapy, I consider removing confounders (summarised in ESM as malfunctioning of ICP monitoring devices, pneumothorax, hypoxia, hypercapnia, pain, hypo/hypertension, hyperpyrexia, seizures, hypo-osmolality). These factors need to be corrected early with specific therapeutical manoeuvres. In all TBI patients, I consider always obtaining an early neurosurgical opinion on surgery for intracranial mass lesions and if the patient presents a clinical or imaging neuroworsening. I will escalate TIL (therapy intensity level) accordingly to the ICP response. The progression I use is summarised in the TILs described below. The therapies with a * are short lasting. TIL 1—Basic. If ICP is > 20–22 mmHg, consider head-up positioning (15–30°), sedation and analgesia: propofol 4–6 mg/kg/h, opioids: fentanyl 1–4 mcg/kg/h), mild hypocapnia* (PaCO_2_ = 35 mmHg), normothermia and antiepileptics (if the patents has seizures or non-convulsive status). Maintain CPP 50–70 mmHg according to autoregulatory status. The risks and level of evidence for these therapies are low but this bundle is effective in many patients for controlling ICP. TIL 2—Mild. If ICP is > 20–25 mmHg with TIL-1 therapies, I consider the following: increasing sedation (side effect: hypotension and need of vasoactive drugs), CSF drainage* inserting external ventricular drainage (side effect: infections, hematoma), osmotherapy* (mannitol and/or hypertonic saline. Maintain a euvolemic status) and mild hypocapnia*. Maintaining CPP 50–70 mmHg according to autoregulatory status. If pressure autoregulation is preserved, higher CPP (around 70 mmHg) is tolerated and might reduce ICP maintaining cerebral blood flow. If pressure autoregulation is not preserved, higher CPP increases cerebral blood volumes and, consequentially, ICP TIL 3—Moderate. If ICP remains > 20–25 mmHg with TIL-2 therapies, I use higher doses of osmotic* (limits: natremia < 155 mEq, Osm 320), profound hypocapnia* with a brain oxygen monitor. CPP 50–70 mmHg according to autoregulatory status. Consider repeating a CT scan. TIL 4—Extreme. If ICP persists > 25 mmHg, refractory to TIL-3 therapies, consider before using extreme therapies the prognosis of the patient, the best outcome that might be obtained and the patient’s wills and inform the patient’s family. Use barbiturates for “buying time” while discussing the utility of decompressive craniectomy. Evaluate DC soon when TIL 3 therapies have failed. I am using moderate hypothermia only in selected cases. See text for details. A continuous check of the efficacy of the therapies needs to be implemented and, if ICP is controlled, consider moving backwards in the flowchart, deescalating ICP lowering as soon as possible

Figure 1 summarises the algorithm that I use in clinical practice. Before starting any ICP-directed therapies, I try to correct any reversible cause and systemic abnormality affecting intracranial volumes and causing raised ICP (see Additional file 1). I always consider the surgical option with a neurosurgeon; mass-occupying space should be promptly evacuated when indications are met, and hydrocephalus should be drained.

When I decide to administer ICP-lowering therapies, I use a “staircase” approach [1] with escalating treatment intensity (starting with low risk-benefit profiles) [8]. The first-line ICP-lowering strategies that I consider (without a priority between them) include:

- Head-up positioning (15–30°),
- Hemodynamic stability aimed to maintain an appropriate cerebral perfusion pressure (CPP 50–70 mmHg according to autoregulatory status. Increasing mean arterial pressure + 10% might be considered as a test for exploring pressure autoregulation),
- Sedation and analgesia (propofol, 4–6 mg/kg/h and opioids, fentanyl 1–4 μg/kg/h used at the lowest dose producing ICP control. Maintain CPP with vasopressors, if needed) [9],
- Mechanical ventilation to prevent hypercapnia and hypoxia (target PaCO_2_ at 35 mmHg, and oxygen saturation ≥ 94%),
- Normothermia; if the temperature is > 37.5 **°**C (internal), I start Diclofenac infusion [10].
- Crystalloids as preferred maintenance fluids [11] to maintain euvolemia and to prevent drops in plasma osmolarity. I do not use colloids or hypotonic solutions w/o glucose as maintenance fluids.

If HICP persists, I subsequently escalate to osmotic agents, mannitol (up to 0.5–1 g/kg every 4–6 h) or hypertonic saline (7.5% solution, 100 ml every 4–6 h). They have several transient mechanisms (lasting 4–6 h) mainly due to osmotic effects but also hemodilution, increased cardiac output and increased blood pressure. I prefer testing both of them (using an equimolar bolus) for evaluating their efficacy in the individual patient. Their efficacy is higher if started at an ICP > 25 mmHg [11].

## When and how to escalate to upper tier therapies

I generally reserve to patients with refractory intracranial hypertension ICP-lowering strategies associated with significant side effects and potential complications as hyperventilation, metabolic suppression and decompressive craniectomy [8, 12].

Hyperventilation produces a reduction of HICP by inducing cerebral vasoconstriction and reducing cerebral blood volume [13]. The effect is short lasting and cease when the interstitial pH, alkalotic during the immediate hyperventilation phase, returns to normality. However, because of the theoretical risk of hypoperfusion, I aim to achieve mild hyperventilation, i.e. a PaCO_2_ ~ 30–32 mmHg, only in patients in whom ICP remains abnormally elevated despite first- and second-line treatments, considering adding for safety a brain oxygenation monitor. I use more aggressive hyperventilation only in life-threatening cases with the risk of cerebral herniation and death.

Barbiturates have been historically used for decreasing brain metabolism and consequently cerebral blood flow/volume and therefore HICP at the cost of serious side effects including hypotension and infections. I avoid long-term administration, and I generally administer thiopentone (10 mg/kg bolus, checking its efficacy, followed by 3–8 mg/kg/h infusion) as temporary “bridge” to decompressive craniectomy (DC) in refractory cases. I prefer, as third tier therapy, DC that has a long-lasting effect on the control of refractory HICP. DC performed without severe refractory HICP increases the rate of unfavourable neurologic outcome and should be avoided [14]. On the other hand, DC in patients with severe refractory HICP reduces mortality (22 more survivors for every 100 patients treated) [15]. At 12 months, 13/22 survivors (59%) had favourable outcomes while 9/22 (41%) were in a vegetative state or in lower severe disability. For these reasons, DC needs to wisely ponder in the context of refractory HICP and it should be undertaken timely in subjects with a potentially acceptable prognosis (i.e. before irreversible damages occurred), considering individual patient’s preferences and family’s quality of life expectations.

In conclusion, my approach to ICP-lowering strategies has a stepwise fashion associated with a continuous check of the efficacy of the therapies. This will allow me to deescalate ICP-lowering strategies as soon as possible (ICP control > 24 h). Tapering therapies (as hyperventilation and osmotic) might produce a rebound effect, and it needs to be done slowly and under ICP monitoring.

Alternatively, if the therapies are ineffective, I intensify treatments until the patients are judged salvable. When, in more severe unsalvageable cases, everything is ineffective and DC is not an option, a wise limitation of the therapies has to be evaluated.

## Additional file

Additional file 1: Summary of the remediable causes of intracranial hypertension. (DOCX 15 kb)

## Data Availability

Not applicable.
