# Supplementary material for: How I manage intracranial hypertension
Source: Crit Care. 2019 Jul 4;23:243. doi: 10.1186/s13054-019-2529-z (PMC6611036; doi:10.1186/s13054-019-2529-z)
Supplement: Supplementary file 1 — Summary of the remediable causes of intracranial hypertension. (DOCX 15 kb) [file 13054_2019_2529_MOESM1_ESM.docx]

ESM1. Remediable causes of intracranial hypertension. Revised from *Maas AIR, Dearden M, Servadei F, Stocchetti N, Unterberg A.* Current recommendations for neurotrauma. Curr Opin Crit Care 2000; 6: 281–292.

Abbreviations: FiO_2_, fraction of inspired oxygen, PaO_2_ partial pressure of oxygen.

| **Cause** | **Mechanism** | **Solution** |
| --- | --- | --- |
| Malfunctioning of ICP monitoring technologies | Calibration errors, zero drift phenomena | Evaluate substitution of the ICP device |
| Intracranial hematoma | Space occupying lesions | Consider neurosurgery.  Ask an opinion to neurosurgeons. |
| Airway obstruction (kinked endotracheal tube, tongue, sputum retention) pneumothorax | Obstruction to venous outflow.  PaCO_2_ rise | Check the tube.  Aspirate secretions.  Search for PNX and eventually drain it |
| Hypoxia | PaO_2_< 60 mmHg can cause secondary hypoxic insult and cerebral vasodilation | Increase FiO_2_ while fixing the cause of hypoxia |
| Hypercapnia (hypoventilation) | Cerebral vasodilation | Increase ventilation (prefer increase ventilatory frequency) |
| Pain, sedation, coughing/straining | Increased metabolism and direct pressure transmission | Optimize sedation |
| Hypotension (hypovolemia, sedation, cardiac) | Reduction of cerebral blood flow if pressure autoregulation is compromised | Evaluate volemia (with echo or other techniques). Infuse fluids if needed and use vasopressors early |
| Hypotension (hypovolemia, sedation, cardiac) | Compensatory cerebral vasodilation related to reduced cerebral perfusion pressure (vasodilatory cascade) if pressure autoregulation is normal | Evaluate volemia (with echo or other techniques).  Infuse fluids if needed and use vasopressors early |
| Hypertension | Increase of cerebral blood flow if pressure autoregulation is compromised | Evaluate sedation adequacy and, eventually, antihypertensive drug |
| Posture (Trendelenburg position, neck rotation) | Reduced venous outflow | Fix the problem with the nurses |
| Hyperpyrexia | Increased cerebral metabolism | Start antipyretics soon |
| Seizures | Increased cerebral metabolism | Monitor and treat seizures promptly |
| Hypo-osmolality (sodium, protein) | Cerebral oedema | Infuse hypertonic infusions |
